# Asperuloside Enhances Taste Perception and Prevents Weight Gain in High-Fat Fed Mice

**DOI:** 10.3389/fendo.2021.615446

**Published:** 2021-04-13

**Authors:** Muhammad Ishaq, Duyen Tran, Yijia Wu, Krzysztof Nowak, Bianca J. Deans, Joycelin Tan Zhu Xin, Hui Lin Loh, Wen Ying Ng, Chin Wen Yee, Benjamin Southam, Silvia Vicenzi, Cameron Randall, Cheng Yang, Ee Tan, Manideepika Pasupuleti, Avneet Kaur Grewal, Tauseef Ahmad, Madhur Shastri, Carmelo Vicario, Maurizio Ronci, Mariachiara Zuccarini, Martin Bleasel, Paul Scowen, William Raffaeli, Gianvicenzo D’Andrea, Dinesh Kumar Chellappan, Glenn Jacobson, Alex C. Bissember, Jason A. Smith, Raj Eri, Juan Canales, Miguel Iglesias, Nuri Guven, Vanni Caruso

**Affiliations:** ^1^ School of Pharmacy and Pharmacology, University of Tasmania, Hobart, TAS, Australia; ^2^ School of Natural Sciences-Chemistry, University of Tasmania, Hobart, TAS, Australia; ^3^ School of Pharmacy, International Medical University, Kuala Lumpur, Malaysia; ^4^ School of Health Sciences, University of Tasmania, Newnham, TAS, Australia; ^5^ Department of Psychology, University of Messina, Messina, Italy; ^6^ Department of Pharmacy, University “G. d’Annunzio”, Chieti, Italy; ^7^ Animal Services department/Research Division, University of Tasmania, Hobart, TAS, Australia; ^8^ Institute for Research on Pain, Istituto di Formazione e Ricerca in Scienze Algologiche (ISAL) Foundation, Rimini, Italy; ^9^ School of Psychological Sciences, University of Tasmania, Hobart, TAS, Australia; ^10^ School of Health Sciences, University of Tasmania, Darlinghurst, NSW, Australia

**Keywords:** asperuloside, CD36, cannabinoid (CB) receptor 1, TAS1R2-3, FFAR1-4, nutrient-sensing mechanisms, food intake, weight loss

## Abstract

Asperuloside is an iridoid glycoside found in many medicinal plants that has produced promising anti-obesity results in animal models. In previous studies, three months of asperuloside administration reduced food intake, body weight, and adipose masses in rats consuming a high fat diet (HFD). However, the mechanisms by which asperuloside exerts its anti-obesity properties were not clarified. Here, we investigated homeostatic and nutrient-sensing mechanisms regulating food intake in mice consuming HFD. We confirmed the anti-obesity properties of asperuloside and, importantly, we identified some mechanisms that could be responsible for its therapeutic effect. Asperuloside reduced body weight and food intake in mice consuming HFD by 10.5 and 12.8% respectively, with no effect on mice eating a standard chow diet. Fasting glucose and plasma insulin were also significantly reduced. Mechanistically, asperuloside significantly reduced hypothalamic mRNA ghrelin, leptin, and pro-opiomelanocortin in mice consuming HFD. The expression of fat lingual receptors (CD36, FFAR1-4), CB1R and sweet lingual receptors (TAS1R2-3) was increased almost 2-fold by the administration of asperuloside. Our findings suggest that asperuloside might exert its therapeutic effects by altering nutrient-sensing receptors in the oral cavity as well as hypothalamic receptors involved in food intake when mice are exposed to obesogenic diets. This signaling pathway is known to influence the subtle hypothalamic equilibrium between energy homeostasis and reward-induced overeating responses. The present pre-clinical study demonstrated that targeting the gustatory system through asperuloside administration could represent a promising and effective new anti-obesity strategy.

## Introduction

Obesity is a complex and multifactorial medical condition characterized by increased adipose tissue stores resulting from a chronic imbalance between energy intake and energy expenditure (1). Genetic and epigenetic signatures as well as many complex behavioral and societal factors contribute to the obesity epidemic in the developed and developing world (2). Long-term prospective follow-up studies have clearly demonstrated that comorbidities associated with obesity including cardiovascular and chronic kidney diseases, diabetes, and some cancers, greatly increase mortality rates in obese patients as well as public healthcare costs (3, 4). Several countries including Australia, USA, UK, and Europe have developed robust guidelines for the short and long-term management of obesity that initially aim to reduce weight through chronic behavior and lifestyle changes and secondly, to utilize pharmacotherapies and/or bariatric surgery (5–7).

Over the last decade, anti-obesity drug discovery programmes have increasingly aimed to identify active compounds from plant sources and traditional medicines as a response to an obesogenic environment that promotes weight gain. Among several emerging therapeutic compounds, the iridoid glycoside asperuloside (ASP), has produced promising anti-obesity results (8). Three months of ASP administration reduced food intake and body weight in rats consuming high-fat diet (HFD) *ad libitum (* 9–11). This compound reduced plasma triglyceride, total cholesterol, and free fatty acid levels as well as circulating levels of glucose and insulin (10). Gene expression studies also indicated that ASP regulates the mRNA levels of enzymes involved in lipid metabolism including a significant downregulation of isocitrate dehydrogenase 3α and fatty acid synthase in the white adipose tissue, an elevation of acyl-CoA dehydrogenase and carnitine palmitoyltransferase levels in the liver, as well as citrate synthase, succinyl CoA synthase and succinate dehydrogenase levels in skeletal muscle (10). However, the mechanisms by which ASP reduces food intake and body weight were not elucidated.

In our study, we attempted to describe a plausible mechanism of action by which ASP induces weight loss. We investigated changes in the taste buds of the tongue as well as orexigenic and anorexigenic markers regulating food intake in the hypothalamus. In fact, beyond the established hypothalamic–mesolimbic pathway circuitry for the regulation of food intake, ingestive behaviors may be regulated by a diverse array of detectors in the oral cavity including taste receptors in the tongue (12–14). Taste buds are located on the dorsal surface of the tongue, on the soft palate, and in the oropharynx (12, 15). They integrate complex chemosensory signaling pathways throughout the gastrointestinal tract leading to endocrine responses affecting gustatory, metabolic, and satiety mechanisms (16, 17). When the taste buds are stimulated by a specific flavor, chemical signals are transmitted to the gustatory center of the brain (i.e. the insular cortex and then hypothalamus) through cranial nerves including the glossopharyngeal nerve, the facial nerve, and the vagus nerve (18). In the obese, the number and density of taste buds is reduced by 25% compared to healthy individuals and this might be associated to overeating due to impairments in afferent reward-induced signaling (19–24). Taste buds are a collection of gustatory sensory cells that release ATP or use neurotransmitters such as acetylcholine, serotonin, norepinephrine, or GABA in response to gustatory stimulation, to communicate within the taste bud itself or with afferent sensory nerves (13). Gustatory cells of the taste buds are divided in four morphological subtypes: Types I, II, III, and IV and among these subtypes, type II cells are the most characterized (12, 25). Type II cells express receptors that are involved in the regulation of ingestive behaviors (14) including the scavenger receptor CD36 (cluster of differentiation 36), a plasma membrane receptor participating in the orosesory detection of dietary lipids (26, 27). Type II cells also express free fatty acid receptor 1 (FFAR1) and free fatty acid receptor 4 (FFAR4) (17). These receptors are known to mediate orosensory responses to long and short-chain fatty acid respectively (12). Orosensory detection of dietary fatty acids is also mediated by the activity of cannabinoid receptor 1 (CB1R) (28) which additionally plays a major role in the enhancement of the sweet taste (29). In addition, the influence of sweet taste sensitivity on food intake is finely regulated by the activity of sweet lingual receptors (TAS1R2-3) whose polymorphisms have been associated with higher consumption of sweet foods (22, 23).

In this study, we aimed to offer a descriptive mechanism of action by which ASP prevented weight gain in animal models (9–11). We investigated changes in the taste buds of the tongue as well as orexigenic and anorexigenic markers regulating food intake in the hypothalamus of obese and lean mice. After seven weeks of treatment ASP selectively limited body weight gain and reduced food intake only in mice consuming HFD, with no effects on mice consuming standard chow diet. Here we propose that ASP exerts its anti-obesity proprieties by altering hypothalamic receptors as well as sweet and fat lingual receptors involved in the regulation of food intake (12, 16, 18, 30).

## Materials and Methods

All animal work was approved by the Animal Care and Ethics Committee of the University of Tasmania (A0015841) and conducted in accordance with the Tasmanian Animal Welfare Act (1993/63) and the Australian Code of Practice (8th Edition 2013) guidelines (31).

### Animals and Diet

Three-week-old C57BL/6J male mice (Animal Services breeding facility, University of Tasmania) were housed at 20 ± 2°C and maintained on a 12:12 h light/dark cycle. After one week of acclimatization, mice (n=40) were assigned to two diet groups: standard chow (12.8 MJ/kg, 6% fat, 20% protein, 3.2% crude fibre, Barastoc, Victoria, Australia) or commercial high-fat pelleted diet (HFD) (19.4 MJ/kg, 23.5% fat, 23% protein, 5.4% crude fibre, Specialty Feeds, Glen Forest, Western Australia) *ad libitum*. Mice were singularly housed, and their body weight and food intake were recorded weekly throughout the study. Mice were euthanized after 12 weeks of ASP administration.

### Preparation of ASP

ASP was extracted and isolated from the native Australian plant *Coprosma quadrifida* (F. Rubiaceae) according to the procedure outlined in Deans et al (32). The isolated crystalline ASP was homogenously mixed in autoclaved water with commercial standard chow powder (Barastoc, Victoria, Australia) and sucrose (4% w/w of food mash). A food pellet of 1 g containing 3 mg of crystalline ASP was served in a small dish in treated animals daily. Control group received a food pellet of 1 g containing a mixture of commercial standard chow powder (Barastoc, Victoria, Australia) and sucrose (4% w/w) daily. All animals consumed all of their 1 g food pellet provided daily.

### Sample Collection

All mice were fasted overnight (12 h), then euthanized *via* carbon dioxide inhalation for tissue and plasma collection. Blood was collected by cardiac puncture and centrifuged (12,000 rpm/8 min). Plasma was separated and stored at −20°C for determination of metabolic markers. Plasma insulin levels were quantified using a commercial Bio-plex pro mouse diabetes kit (Cat#171F7001M, Bio-Rad, Australia) according to the manufacturer’s instructions. After decapitation, the cortex and the ventral region of hypothalamus containing the arcuate nucleus were removed, frozen in liquid nitrogen then stored at −80°C for determination of gene expression. The tongue and liver were dissected and frozen in liquid nitrogen and stored at −80°C for mRNA expression. Visceral fat pads (epididymal and retroperitoneal fat) and interscapular brown adipose tissue (IBAT) were dissected, weighed and stored at −80°C for future investigations, as were skeletal muscles (anterior tibialis and gastrocnemius).

### RNA Extraction and cDNA Synthesis

Total RNA was extracted and purified from frozen tissues using RNeasy Mini kit (Cat# 74104, Qiagen, Japan) from individual samples according to the manufacturer’s recommendations and stored at −80°C for further processing. A Nanodrop™ 8000 Spectrophotometer (NanoDrop Technologies Inc., Japan) was used to measure the RNA concentration and purity ratios (A260/280 and A260/230) and only samples with absorbance ratio of ~2.0 were used for complementary DNA (cDNA) synthesis. One microgram of RNA template from each sample was reverse transcribed into cDNA using iScript™ Reverse Transcription Supermix (Cat# 1708840, Bio-Rad, Australia) in a final reaction volume adjusted to 20 µl.

### Primer Designing and Quantitative Real-Time PCR (qRT-PCR)

All primers were designed using PrimerQuest tool (Integrated DNA Technologies, Inc., USA). For gene transcription, the primers used were ghrelin, leptin receptor, proopiomelanocortin (POMC), cannabinoid receptor type 1 (CB1R), taste receptor type 1 member 2 (TAS1R2), taste receptor type 1 member 3 (TAS1R3), free fatty acid receptor 1 (FFAR1) and free fatty acid receptor 4 (FFAR4). The sequences of custom-designed primers are in the **Supplementary Table**. Primers optimization was carried out with a gradient qPCR (52–62°C) to achieve the best melting temperature and efficiency for amplifying the target genes. qPCR reactions were carried out in duplicates using SsoAdvanced™ Universal Inhibitor-tolerant SYBR green supermix (Cat # 1725017, Bio-Rad, Australia) in a QuantStudio™ 3 real-time PCR system (Thermo Fisher Scientific, Australia) following manufacturer’s instructions. Each qPCR reaction was carried out in the final volume of 10 μl, with a concentration of 2.5 ng/µl for cDNA and 400 µM for primers. The amplification program included an initial denaturation step at 98°C for 3 min followed by 40 cycles of 10 s at 98°C, 12 s at 54–60°C (optimal temperature for each primer pair) and 20 s at 72°C. After each amplification, a melting curve study was conducted to validate the product specificity. In order to ensure the reliability of the results, the raw Ct-values were compared, and the outlier’s samples with a standard deviation higher than 0.3 or 0.5 Ct cycle were removed. A GeNorm software analysis was performed to calculate the stability of the housekeeping genes among different treatment groups while RefFinder algorithm was used to produce comprehensive ranking (33). Target gene expression was normalized against b-actin and ribosomal protein 19 housekeeping genes using a sample from the control group as calibrator. All the analysis was performed using the comparative ΔΔCt method (34).

### Statistical Analysis

Statistical analysis was performed using GraphPad Prism version 8.3.0 for Windows (GraphPad Software, San Diego, California USA, www.graphpad.com) and results were expressed as mean ± SEM.

The effects of asperuloside on body weight and energy expenditure were analysed by Repeated Measures three-way ANOVA with animal diet, drug treatment, and weeks of treatment as factors. The effects of asperuloside on final body weight, food daily intake, visceral fat, glucose, and plasma insulin concentrations, hypothalamic and lingual gene expressions were analyzed by two-way ANOVA with animal diet and drug treatment as factors. ANOVA results were then followed by a *post hoc* analysis using Fisher’s least significant difference test (LSD) as appropriate. Results were considered statistically significant when *p* < 0.05.

## Results

### Effect of ASP on Body Weight, Energy Intake, Adiposity, Blood Glucose, and Plasma Insulin

**Figure 1:**
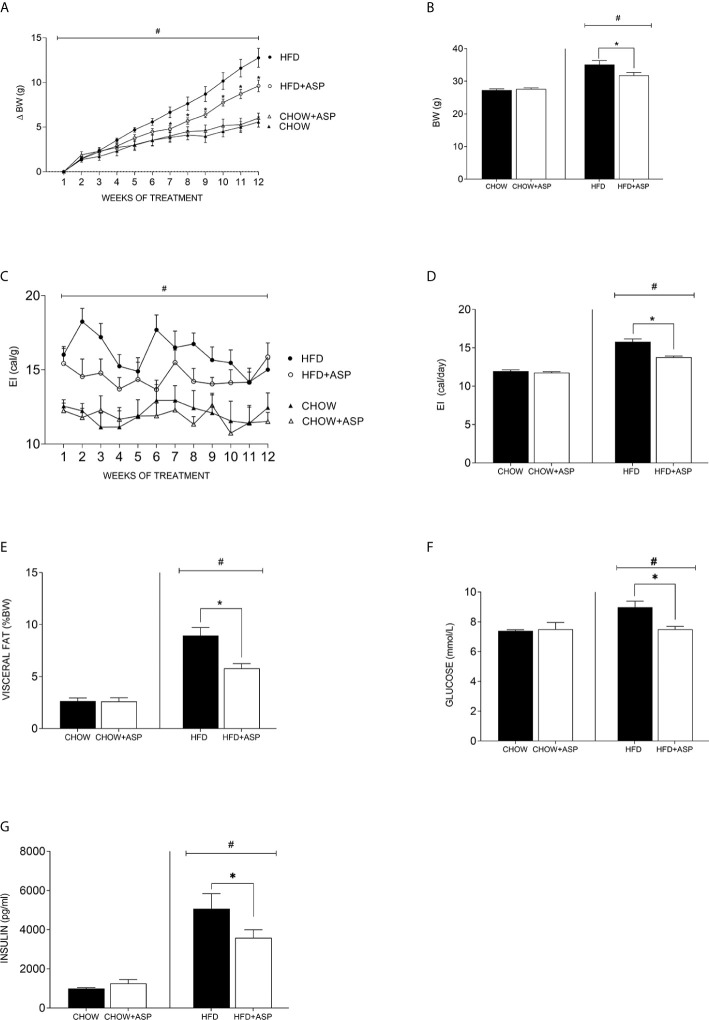
Effect of asperuloside on body weight, fat content, glucose, and insulin. **(A)** Change in body weight (δBW) of mice consuming standard chow diet (CHOW) (black triangle, n=10), and those treated with asperuloside (ASP) (open triangle, n=10); mice consuming high fat diet (HFD) (black circle, n=10), and treated with ASP (open circle, n=10). **(B)** Final body weight after 12 weeks of ASP administration (n=10). **(C)** Energy intake (EI) of mice consuming standard chow diet (black triangle, n=10), and treated with ASP (open triangle, n=10); mice consuming HFD (black circle, n=10), and treated with ASP (open circle, n=10). **(D)** Average of daily EI (n=10). **(E)** Visceral fat (epididymal and retroperitoneal fat pads) at killing calculated as percentage of wet weight to body weight (n=10). **(F)** Fasting blood glucose (n=10); **(G)** Plasma Insulin (n= 6) after 12 weeks of asperuloside administration. Results are expressed as mean ± SEM. In graphs **(A, C)** data were analyzed by Repeated Measures three-way ANOVA with animal diet, drug treatment and weeks of treatment as factors. In graphs **(B, D–G)** data were analyzed by two-way ANOVA with animal diet and drug treatment as factors. ANOVA results were then followed by a *post hoc* analysis using Fisher’s least significant difference test (LSD). *Significant ASP effect vs non-treated diet control (p < 0.05); ^#^Significant overall diet effect (HFD and HFD+ASP) vs (CHOW and CHOW+ASP) (p < 0.05).

Mice eating high fat diet (HFD) had greater body weight (BW) gain over the experimental period of 12 weeks compared to standard chow diet group [*F*(3, 35) = 9.010; *p*<0.05; **Figure 1A**].

ASP (3 mg/day) only significantly reduced BW in mice consuming HFD [−10.50%; *F*(3, 36) = 22.50; *p*<0.05; **Figures 1A, B**]. Weight loss reached significance after seven weeks of treatment and was maintained until the end of the experiment (**Figure 1A**).

Over the experimental period, ASP did not impact the daily or cumulative energy intake (EI) of mice on standard chow (**Figures 1C, D**). In contrast, in mice consuming HFD ASP significantly reduced daily EI when compared to the control [−12.8%; *F*(3, 52) = 71.25; *p*<0.05, **Figure 1D**].

Significant effects of ASP were also observed across several adipose tissue depots including visceral fat pads (epididymal and retroperitoneal fat) only in mice consuming HFD. When standardized by body weight, HFD increased visceral adipose mass (epididymal and retroperitoneal fat pads) and this effect was significantly reduced by ASP treatment [−35%; *F*(3, 36) = 35.38; *p*<0.05, **Figure 1E**]. ASP did not reduce visceral adipose mass in mice eating a standard chow diet. Overall, HFD increased fasting glucose levels compared to standard chow diet (HFD and HFD+ASP) vs (CHOW and CHOW+ASP). ASP significantly reduced those levels only in mice consuming HFD [n=10; −16%; *F*(3, 20) = 3.036; *p*<0.05; **Figure 1F**].

ASP did not affect plasma insulin levels of mice eating standard chow diet (**Figure 1G**). Overall, HFD increased plasma insulin level compared to standard chow diet (HFD and HFD+ASP) vs (CHOW and CHOW+ASP) and after 12 weeks of drug administration, ASP significantly reduced these levels [n=6; −29%; *F*(3, 15) = 19.99; *p*<0.05; **Figure 1G**].

### Effects of ASP on Sweet and Fat Taste Receptors of the Tongue

**Figure 2:**
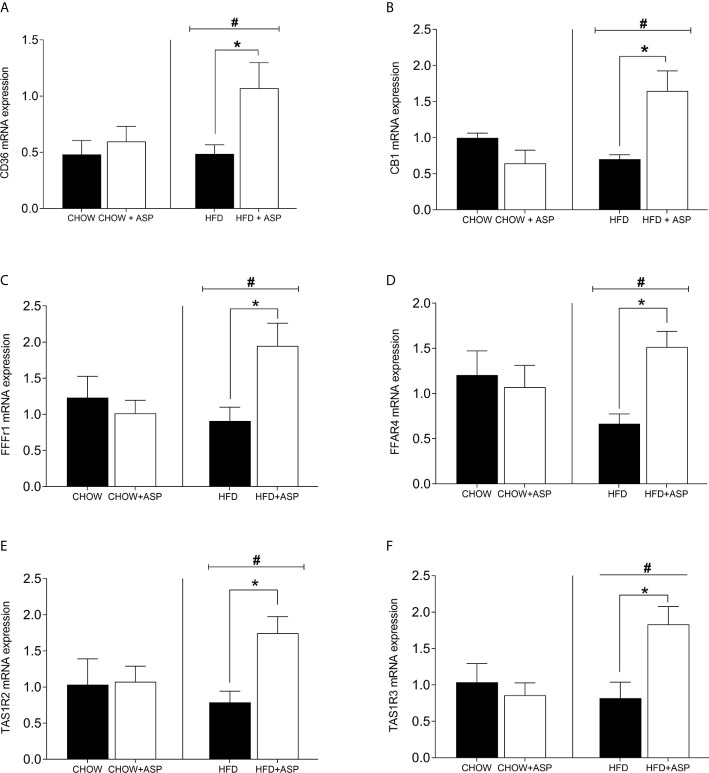
mRNA levels of taste receptors in the tongue. **(A)** Cluster of differentiation 36 (CD36); **(B)** Cannabinoid receptor 1 (CB1R); **(C)** Free fatty acid receptor 1 (FFAR1); **(D)** Free fatty acid receptor 4 (FFAR4); **(E)** Taste receptor type 1 member 2 (TAS1R2); **(F)** Taste receptor type 1 member 3 (TAS1R3). n=6–9. High fat diet (HFD), asperuloside (ASP). Results are expressed as mean ± SEM. Data were analyzed by two-way ANOVA with animal diet and drug treatment as factors. ANOVA results were then followed by a *post hoc* analysis using Fisher’s least significant difference test (LSD). *Significant ASP effect vs non-treated diet control (p < 0.05); ^#^Significant overall diet effect (HFD and HFD+ASP) vs (CHOW and CHOW+ASP) (p < 0.05).

In mice consuming HFD, the mRNA expression levels of cluster of differentiation 36 (CD36) [n=6; F(3, 14) = 4.84; p<0.05], Taste 1 member 2 (TAS1R2) sweet receptors [n=7; F(3, 15) = 4.08; p<0.05], Taste 1 member 3 (TAS1R3) [n=6–7; F(3, 14) = 9.29; p<0.05] as well as Free fatty acid receptor 1 (FFAR1) [n=6; F(3, 14) = 4.69; p<0.05] and Free fatty acid receptor 4 (FFAR4) [n=6; F(3, 14) = 3.23; p<0.05] was nearly doubled after after 12 weeks of ASP administration. Overall, HFD increased the expression levels of these genes compared to standard chow diet (HFD and HFD+ASP) vs (CHOW and CHOW+ASP) (**Figure 2**). The compound did not alter the lingual mRNA expression of the abovementioned genes in mice consuming a standard chow diet.

### Effects of ASP on Hypothalamic Genes

**Figure 3:**
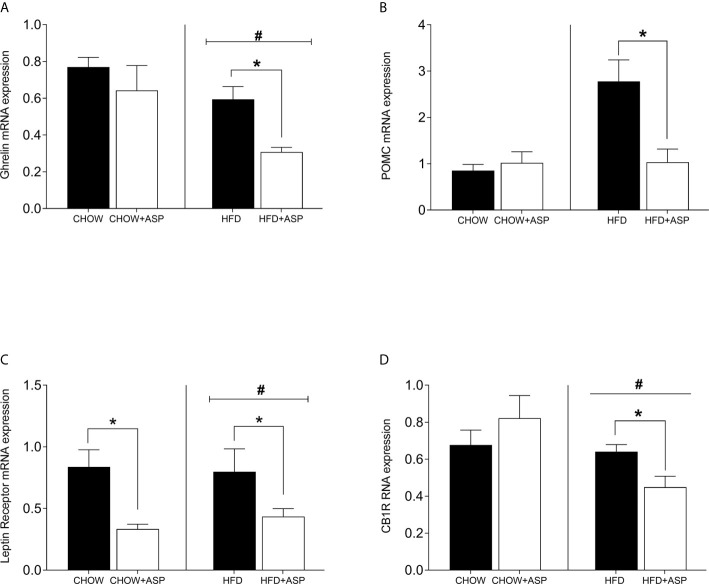
Hypothalamic mRNA levels of orexigenic and anorexigenic markers consuming HFD. **(A)** Ghrelin receptor **(B)** Pro-opiomelanocortin (POMC); **(C)** Leptin receptor; **(D)** Cannabinoid 1 receptor (CB1) n=6–9. High fat diet (HFD), asperuloside (ASP). Results are expressed as mean ± SEM. Data were analyzed by two-way ANOVA with animal diet and drug treatment as factors. ANOVA results were then followed by a *post hoc* analysis using Fisher’s least significant difference test (LSD). *Significant ASP effect vs non-treated diet control (p < 0.05); ^#^Significant overall diet effect (HFD and HFD+ASP) vs (CHOW and CHOW+ASP) (p < 0.05).

In mice consuming HFD, 12 weeks of ASP administration significantly reduced the expression of hypothalamic markers including ghrelin [n=7–8; F(3, 20) = 4.15; p<0.05] and pro-opiomelanocortin (POMC) [n=6–7; F(3, 15) = 7.242; p<0.05]. ASP did not alter the hypothalamic mRNA expression of mice exposed to a standard diet with the only exception of leptin receptor [n=6–7; F(3, 16) = 4.11; p<0.05]. Overall, HFD increased the expression levels of these genes compared to standard chow diet (HFD and HFD+ASP) vs (CHOW and CHOW+ASP) (**Figure 3**).

## Discussion

The present study identified mechanisms that could be responsible for anti-obesity properties of Asperuloside (ASP), while confirming previously reported anti-obesity properties (9–11). Over the 12 weeks of experimental period, oral ASP administration (3mg/day) induced a significant reduction in food intake and body weight in only mice consuming HFD compared to their control group. Weight loss reached significance after seven weeks of treatment and was maintained until the end of the experiment with a final body weight difference of 10.5% between treated and untreated animals.

In the HFD group, ASP also induced a 12.8% reduction in daily energy intake compared to the control group, while in mice eating a standard chow diet, body weight and daily or cumulative energy intake were not affected by ASP. ASP promoted a significant reduction of visceral adipose mass (epididymal and retroperitoneal fat pads) as well as blood glucose and insulin levels in mice consuming HFD.

In our study, ASP had no effect on body weight, adipose mass, daily or cumulative energy intake, and on the hypothalamic and lingual mRNA levels of mice on a standard chow diet. For these reasons, the present discussion focuses only on the effects of ASP in mice consuming HFD.

**Figure 4:**
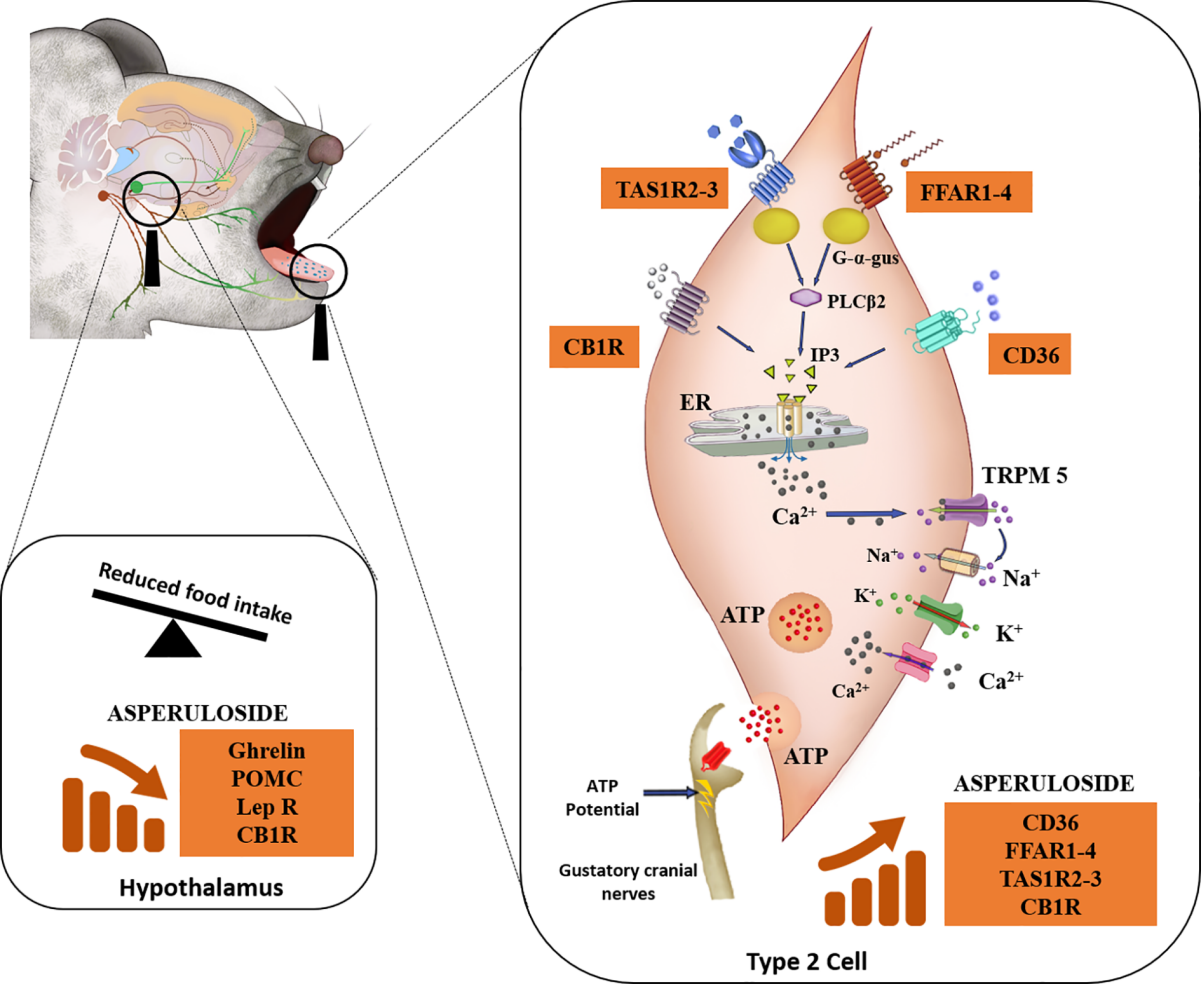
Descriptive mechanism of action of ASP. Mice consuming either a standard chow diet or HFD received 3 mg of ASP daily for 12 weeks. We hypothesize that ASP prevented weight gain in mice consuming HFD by altering nutrient-sensing mechanisms of the taste buds that are involved in the homeostatic and hedonic regulation of food intake (12, 14, 16, 18, 30, 35). In the taste buds, gustatory Type II cells express the plasma membrane receptor CD36 (cluster of differentiation 36), a receptor participating in the orosesory detection of dietary lipids (26). Type II cells also express the free fatty acid receptor 1 (FFAR1) and free fatty acid receptor 4 (FFAR4) which mediate orosensory responses to long and short-chain fatty acid respectively (12). They also express subfamilies of the taste receptor type 1 (TAS1R) which detect sweet, bitter, and the umami taste (16). The endocannabinoid system is also involved in the orosensory detection of dietary fatty acids (14). This physiological function is mediated by the activity of cannabinoid receptor 1 (CB1R) (28) which additionally plays a major role in the enhancement of the sweet taste (29). When ASP was administered in mice consuming HFD, levels of CB1R, sweet (TAS1R2 and TAS1R3) and fat (CD36, FFAR1, and FFAR4) receptors were nearly doubled. FFAR1-4 and TAS1R2-3- are coupled to the G-protein α-gustducin (G-α-gus) which activates phospholipase C-β2 to promote the formation of inositol triphosphate (IP3) and the final release of intracellular Ca^2+^ from the endoplasmic reticulum (ER) (14, 17, 36). Similarly, when dietary lipids bind to CD36, this will ultimately result in the rise IP3 (14). The binding of IP3 to ER will release free Ca^2+^, followed by Ca2^+^ efflux (14). The increase in intracellular Ca^2+^ activates the transient receptor potential cation channel M5 (TRPM5) and then the voltage-gated Na^+^ channels allowing the entrance of Na^+^ ions and the outward current of K^+^ ions, inducing membrane depolarisation as well as ATP release (12, 16). At the same time, neurotransmitters and peptides involved in the regulation of food intake including NPY, ghrelin, and leptin are released to modulate autocrine and paracrine signaling within the taste bud itself and with afferent sensory nerves (13, 16). In response to taste stimulation, ATP release is increased into the cranial nerves VII and/or X and this elicits a greater afferent nutrient-sensing signal to the brain (12, 13, 17, 18, 35, 37). Taste signaling increases cortical activity in the mesolimbic pathway (regions of the brain associated with hedonic responses) which in turn send chemical signals to the hypothalamus for the regulation of food intake (35, 36). In the hypothalamus of mice consuming HFD, ASP downregulated the gene expression of appetite regulators including ghrelin, POMC, leptin, and CB1R (36, 38, 39). We hypothesize that ASP overstimulates taste buds signaling in response to high fat diet consumption leading to suppression of orexigenic signaling in the hypothalamus (12, 16, 18, 30, 35).

In addition to previous studies investigating the anti-obesity properties of ASP (9–11), we aimed to describe a possible mechanism of action for ASP. Our results suggest that ASP might exert its therapeutic effect by altering fat and sweet receptors in the oral cavity, which are known to affect appetite, satiety, and metabolism through afferent signaling to the hypothalamus, brain area that regulates energy homeostatic responses to nutrient utilisation (12) (**Figure 4**).

Food intake and ingestive behaviors are synergistically regulated by a diverse array of detectors in the oral cavity including taste receptors on the tongue (12). The taste system activates complex chemosensory signaling pathways throughout the gastrointestinal tract leading to endocrine responses affecting gustatory, metabolic, and satiety mechanisms (16). For instance, it is demonstrated that the endocannabinoid system enhances central orexigenic mechanisms and promotes consumption of palatable food (38). Interestingly, recent evidences also confirmed its physiological involved in the peripheral regulation of the gustatory system (14). In fact, the activation of lingual CB1R enhanced sweet taste perception (29) and the mediation of fat taste signaling (28). Conversely, leptin which promotes central anorexigenic signals, acts on peripheral leptin receptors of taste cells to suppress sweet responses, without modifying responses to sour, salty, and bitter substances (30).

On the basis of this recent scientific literature, we tested the effect of ASP on the mRNA expression fat taste receptors (CD36, FFAR1-4), sweet taste receptors (TAS1R2-3) as well as CB1 on the tongue of mice consuming either HFD or chow diet (**Figure 2**).

Fat taste sensitivity is decreased in obesity (14). Obese patients who carry a single nucleotide polymorphism (rs1761667) in the CD36 gene exibited lower CD36 expression associated with lower oral detection for lipids and this might contribute to development of obesity (24). CD36 binds to saturated and unsaturated long-chain fatty acids (LCFA) with an affinity in the nanomolar range (40). It has to be noted that in rodents, lingual CD36 expression is upregulated in a pre-meal situation following a prolonged fast, and it gradually decreases secondary to the food intake (14, 41). On the other hand, the presence of a second receptor (FFRA1) for the same taste suggests that the orosensory detection of dietary lipids could be regulated by the feeding state, fat content of the diet or by other still unclear mechanisms (14). Recent studies confirmed that while CD36 is primarily implicated in the oral detection of dietary lipids (42), FFAR1 signaling is involved in the regulation of post-ingestive behaviors (14, 43). Our results demonstrate for the first time that ASP nearly doubled the expression of CD36, FFAR1, and FFAR4 in animal consuming HFD, but it did not alter their expression in mice eating chow diet. This is in line with previous studies showing that rodents have differential satiety responses to diets rich in fat or carbohydrates due to the receptor variability of the taste buds (44).

As genetic variability, sensorial perceptions, and signaling from other taste receptors could influence food preference and consumption between individuals (45), we complemented the investigation of fat receptors with the investigation of sweet taste receptors TAS1R2, TAS1R3, and CB1R (23, 38). The involvement of hypothalamic CB1R activity in the regulation of food intake is well documented (46). Interestingly, CB1R is also expressed in the tongue (29) making its peripheral contribution in gustatory perception under scrutiny. Specifically, CB1R is expressed in type II taste cells which also express the TAS1R3 sweet taste receptors (25). Scientific evidences demonstrated that endocannabinoids selectively enhance sweet taste by acting on tongue taste cells and that this effect could be mediated by CB1R (29). The latest and most specific evidences regarding the role of endocannabinoid system in the regulation of gustatory signaling arise from physiological studies at the receptor level using transgenic mice and synthetic ligands Investigations on CB1R^−/−^ mice revealed that deletion of the endocannabinoid receptor led to loss of spontaneous preference for fat solutions compared to wild type (28). In line with these findings, ASP increased significantly the lingual expression of CB1R as well as TAS1R2 and TAS1R3 providing a direct evidence that the compound could affect the gustatory signaling mediated by both dietary fatty acids and sweet intake (28). Thus, the greater reduction in food intake we observed in mice consuming HFD and treated with ASP could be associated with the marked alterations in their taste receptors. ASP might stimulate taste buds signaling leading to the release of anorexigenic peptides into intragemmal fibres of afferent taste nerves (35) that in turn might promote orexigenic signaling to the hypothalamus (13, 18, 35) (**Figure 4**). Specifically, as ASP administration impacted the gustatory gene expression only in mice consuming HFD, we hypothesize that the compound might contribute to the activation of orosensory pathways in the reward areas of the brain which in turn enhance satiety and reduce food intake (45). Further studies will be needed to fully elucidate the role of ASP in hedonic areas of the brain regulating food intake.

However, based on mRNA changes in the taste buds, which are known to regulate important functional roles in signaling afferent sensory nerves in the central nervous system (12, 18, 35, 47), we investigated the effects of ASP on the expression of major hypothalamic peptides and receptors regulating food intake (**Figure 3**).

Over the last two decades, several functional studies in animals have established the importance of melanocortin signaling in the regulation of energy homeostasis and food intake, among other important functions (48, 49). It has been demonstrated that oral and intracerebroventricular injections (ICV) of melanocortin-4 receptor (MC4R) antagonists stimulate feeding in satiated rats (48, 50, 51) and that the anorexigenic hormone leptin stimulates the pre-hormone POMC to mediate MC4R signaling in the regulation of food intake and energy expenditure (48, 52, 53). In our study, downregulation of hypothalamic POMC as well as leptin receptor might explain, or at least in part, the reduction in food intake and body weight we observed in mice receiving ASP. When animals are starved, as they were in our experiment, hormone leptin levels decrease, the activity of POMC neurons decrease, and this results in decreased melanocortin signaling (48). Studies also show that melanocortin signaling could be also inhibited by the activity of ghrelin, a hormone mainly produced in the stomach during fasting or dietary restrictions (54). Ghrelin enables orexigenic impulses, interfering with the activity of the anorexigenic peptide α-melanocyte stimulating hormone (α-MSH), the cleaved product of POMC (55). Taken together, ASP significantly downregulated POMC and leptin receptor during starvation suggesting that the compound could exert a synergistic role with leptin in the regulation of food intake.

Another important hormone for the regulation of energy balance is insulin. While systemic injections of insulin are associated with body weight gain in humans (56), animal models have demonstrated that ICV insulin injections reduce food intake, downregulate hypothalamic NPY and increase POMC expression (57), thus exerting the satiety properties of insulin in the fed state. Furthermore, diminished hypothalamic insulin signaling results in a rapid onset of hyperphagia and increased fat mass sufficient to promote obesity and induce peripheral insulin resistance (58). In our study, ASP significantly decreased plasma insulin levels as well as hypothalamic mRNA levels of insulin receptor and neuropeptide Y (NPY). This was accompanied by a decrease in glucose levels, profile commonly associated with an increase in insulin sensitivity (59). While direct evidence is lacking, ASP could promote hypothalamic insulin sensitivity leading to increased satiety despite reduced food intake, mechanism demonstrated after central administration of metformin, first-line agent for type 2 diabetes (60, 61).

Current drug discovery has been under mounting pressure to identify potential compounds targeting the endocannabinoid system. This is in light of the fact that cannabinoid type 1 receptors have been shown to control lipid (28) and glucose metabolism (46) and participate in the regulation of food intake, energy expenditure as well as thermogenesis (62). In our study, although it only approached statistical significance (p=0.069), ASP reduced hypothalamic CB1R mRNA expression in mice consuming HFD suggesting that it could be one such compound. However, the relationship between downregulation of hypothalamic CB1R and the anti-obesity effects of ASP in relation to food intake and body weight reduction needs to be fully elucidated.

Overall, based on the data presented in this study, we propose a descriptive action by which ASP might overstimulate taste bud signaling in response to the consumption of a high fat diet, resulting in the suppression of orexigenic signaling in the hypothalamus (**Figure 4**). To address limitations of our study, high performance liquid chromatography (HPLC) investigations would clarify the pharmacodynamic properties of the compound and whether it exerts its therapeutic effects through a direct central mechanism or *via* peripheral hedonic stimuli. In addition, the integration of previous (9, 10) and our findings with a “multi-omics” approach including genomics, proteomics, and metabolomics would represent a powerful adjunctive tool to understand the mechanistic breadth of action of ASP. The current study did not decipher the contribution of specific neuronal populations with regards to the mechanisms of action of ASP. In fact, exploring the homeostatic (energy demand) versus the hedonic (reinforcing and rewarding aspects of food) mechanisms regulating feeding behavior (39) should occur in animal models in which neuronal populations are selectively ablated (63).

There is a consensus that purinergic signaling is highly involved in the regulation of orogustatory mechanisms (17, 37). Functional studies in animal models have produced exciting discoveries on the role of purinergic signaling in the regulation of food intake. For instance, stimulation of purinergic receptors in the nucleus accumbens (NAc), a primary site mediating reward food behavior, reinforced dopaminergic responses and enhanced food intake (64), while the blockade of these receptors decreased the feeding responses associated with dopamine release (65). Investigating the role of ASP within purinergic signaling might contribute to decipher its mechanism of action contributing to the reduction in food intake we observed in our experiment.

In summary, the present study attempts to provide a descriptive indication by which ASP prevents weight gain when exposed to HFD consumption. While we confirmed previous finding regarding the anti-obesity properties of ASP (9–11), our results indicate that the therapeutical effect of ASP likely occurs *via* alterations in nutrient-sensing mechanisms of the taste buds, which are involved in the homeostatic and hedonic regulation of food intake through afferent signaling to the hypothalamus (12, 14, 16, 18, 30, 35). To date, anti-obesity agents targeting signaling pathways in metabolic tissues such as liver, adipocytes, and skeletal muscles, have failed to deliver significant clinical results (66). The present pre-clinical study demonstrated that targeting the gustatory signaling pathways could represent a promising and effective new anti-obesity strategy. This unique and novel mechanism of action that targets peripheral sensory pathways of the gustatory system should be exploited in future drug discovery programmes to identify new drug candidates that can provide clinically relevant anti-obesity agents.

## Data Availability Statement

The raw data supporting the conclusions of this article will be made available by the authors, without undue reservation, to any qualified researcher.

## Ethics Statement

The animal study was reviewed and approved by Animal Ethics Committee University of Tasmania A0015841.

## Author Contributions

This study was designed and coordinated by VC. The compound was extracted and isolated by BD, AB, and JS. Animal work was performed by MIs, KN, TA, and MS. Tissue collection was performed by MIg, KN, WN, CWY, SV, CY, TA, MS, MB, PS, and VC. Laboratory investigations and statistical analyses were performed by MIs, DT, YW, KN, JT, HL, WN, CWY, BS, SV, CY, ET, MP, AG, TA, MS, MB, PS, and VC. Manuscript was designed and prepared by VC. Manuscript was revised and commented by MIs, DT, BD, SV, CR, CV, MR, MZ, GD, WR, MB, PS, DC, GJ, AB, JS, RE, JC, MI, and NG.

## Conflict of Interest

The authors declare that the research was conducted in the absence of any commercial or financial relationships that could be construed as a potential conflict of interest.
