# Supplementary material for: Asperuloside Enhances Taste Perception and Prevents Weight Gain in High-Fat Fed Mice
Source: Front Endocrinol (Lausanne). 2021 Apr 13;12:615446. doi: 10.3389/fendo.2021.615446 (PMC8076851; doi:10.3389/fendo.2021.615446)
Supplement: Supplementary file 1 [file Table_1.docx]

| Accession no | Transcript | 5’ Forward 3’ | 5’ Reverse 3’ |
| --- | --- | --- | --- |
| NM_021488.5 | GHRL | TGGAGATCGCGCAGATCAG | CCGGGAACTCTCATCCTTCAG |
| NM_146146.3 | LEPR | AAATGTCACCTTGCTTTGGAAGCC | ACGATGCTTCACCACGTACCTC |
| 10336022-350288 A04 | CB1R | AAGTCGATCTTAGACGGCCTT | TCCTAATTTGGATGCCATGTCTC |
| NM_008895.3 | POMC | GCCACTGAACATCTTTGTCCC | AATCTCGGCATCTTCCACGT |
| NM_010568 | INSR 1 | GGTGAACCTCAGTCCTAAC | GAGAAAGTCTCGGAGCTATG |
| NM_031103 | RPL19 | TCGCCAATGCCAACTCTCGTC | AGCCCGGGAATGGACAGTCAC |
| NM_031144.3 | Actb | CACTGCCGCATCCTCTTCCT | AACCGCTCATTGCCGATAGTG |

Supplementary table 1. Hypothalamic and lingual primer sequences

| NM_031873 | TAS1R2 F | CCTTCCAAAGCATCGCCTCCTA | CCAGGCTGGCAACTCTTAGAAC |
| --- | --- | --- | --- |
| NM_031872 | TAS1R3 F | TACAGTGTGGCTCAAGCCCTTC | CAAACTGTAGTGTCAAGTCTCGAG |
| NM_001159558 | FAT/CD36 | GATGACGTGGCAAAGAACAG | TCCTCGGGGTCCTGAGTTAT |
| NM_194057 | Ffar1 F | GCTTGGTCTACACTCTCCATCTG | CCAAGGCAAAGACTGGGCAGAA |
| NM_181748 | Ffar4 F | GTGACTTTGAACTTCCTGGTGCC | CAGAGTATGCCAAGCTCAGCGT |
